# Federal Retail Pharmacy Program Contributions to Bivalent mRNA COVID-19 Vaccinations Across Sociodemographic Characteristics — United States, September 1, 2022–September 30, 2023

**DOI:** 10.15585/mmwr.mm7313a2

**Published:** 2024-04-04

**Authors:** Roua El Kalach, Nkenge Jones-Jack, Mattie A. Elam, Abdulhakeem Olorukooba, Marley Vazquez, Shannon Stokley, Sarah Meyer, Sunanda McGarvey, Kimvy Nguyen, Lynn Gibbs Scharf, LaTreace Q. Harris, Christopher Duggar, Lori B. Moore

**Affiliations:** ^1^Immunization Services Division, National Center for Immunization and Respiratory Diseases, CDC; ^2^Goldbelt Professional Services, LLC, Chesapeake, Virginia; ^3^Peraton, Reston, Virginia.

**Summary**

**What is already known about this topic?**

Pharmacies participating in the Federal Retail Pharmacy Program (FRPP) served as integral partners in national efforts to scale up vaccination capacity during the COVID-19 pandemic emergency response.

**What is added by this report?**

Among 59.8 million COVID-19 bivalent vaccine doses administered in the United States during September 1, 2022–September 30, 2023, 40.5 million (67.7%) were administered by FRPP partners. In urban and rural areas, FRPP partners administered 81.6% and 60.0% of bivalent vaccine doses, respectively.

**What are the implications for public health practice?**

FRPP partnerships were critical in ensuring access to bivalent COVID-19 vaccination services in the United States and could serve as a model to address vaccination services needs for routine vaccines and during future responses to vaccine-preventable disease emergencies.

## Abstract

The Federal Retail Pharmacy Program (FRPP) facilitated integration of pharmacies as partners in national efforts to scale up vaccination capacity during the COVID-19 pandemic emergency response. To evaluate FRPP’s contribution to vaccination efforts across various sociodemographic groups, data on COVID-19 bivalent mRNA vaccine doses administered during September 1, 2022–September 30, 2023, were evaluated from two sources: 1) FRPP data reported directly to CDC and 2) jurisdictional immunization information systems data reported to CDC from all 50 states, the District of Columbia, U.S. territories, and freely associated states. Among 59.8 million COVID-19 bivalent vaccine doses administered in the United States during this period, 40.5 million (67.7%) were administered by FRPP partners. The proportion of COVID-19 bivalent doses administered by FRPP partners ranged from 5.9% among children aged 6 months–4 years to 70.6% among adults aged 18–49 years. Among some racial and ethnic minority groups (e.g., Hispanic or Latino, non-Hispanic Black or African American, non-Hispanic Native Hawaiian or other Pacific Islander, and non-Hispanic Asian persons), ≥45% of COVID-19 bivalent vaccine doses were administered by FRPP partners. Further, in urban and rural areas, FRPP partners administered 81.6% and 60.0% of bivalent vaccine doses, respectively. The FRPP partnership administered approximately two thirds of all bivalent COVID-19 vaccine doses in the United States and provided vaccine access for persons across a wide range of sociodemographic groups, demonstrating that this program could serve as a model to address vaccination services needs for routine vaccines and to provide health services in other public health emergencies.

## Introduction

Approximately 90% of U.S. residents live within 5 miles (8 km) of a community pharmacy, making pharmacies a highly accessible health care resource, particularly among low-income communities (*1*). Understanding the role that pharmacies played during the COVID-19 response is important for future public health planning. The Federal Retail Pharmacy Program (FRPP), a collaboration between the federal government, U.S. states and territories, and 21 national pharmacy chains and independent pharmacy networks, was established to ensure broad access to COVID-19 vaccines (*2*). Since February 11, 2021, participating pharmacies have provided access to COVID-19 vaccines across all 50 states, the District of Columbia (D.C.), U.S. territories, and freely associated states (*2*). On September 1, 2022, CDC recommended COVID-19 bivalent boosters for persons aged ≥12 years (*3*). On October 12, 2022, the recommendation was expanded to include children aged 5–11 years, and on December 9, 2022, children aged 6 months–4 years (*3*). COVID-19 mRNA bivalent vaccine doses reported to CDC by FRPP partners and all bivalent vaccine doses administered in the United States during September 1, 2022–September 30, 2023, were assessed across sociodemographic groups to learn more about FRPP’s contribution to COVID-19 bivalent vaccination.

## Methods

The proportion of COVID-19 bivalent vaccine doses administered in the United States by FRPP partners was calculated using two independent data sources: 1) FRPP bivalent dose administration data reported directly to CDC and 2) all-provider (i.e., FRPP and non-FRPP vaccine providers) data on bivalent vaccine dose administration submitted to each jurisdiction’s immunization information system,* which were then submitted to CDC by each jurisdiction,^†^^,^^§^ for doses administered from September 1, 2022 (the date CDC first issued the recommendation for COVID-19 bivalent vaccination for persons aged ≥12 years) (*3*), through September 30, 2023. Data were analyzed across six age cohorts (6 months–4 years, and 5–11, 12–17, 18–49, 50–64, and ≥65 years), sex, seven categories of race and ethnicity (Hispanic or Latino, non-Hispanic American Indian or Alaska Native [AI/AN], non-Hispanic Asian [Asian], non-Hispanic Black or African American, non-Hispanic Native Hawaiian or other Pacific Islander, non-Hispanic other [other], and non-Hispanic White [White]), and urban-rural classification,^¶^^,^** based on the county where the vaccine dose was administered. Data were analyzed using Microsoft SQL Server Management Studio (version 18; Microsoft) and are descriptive in nature. This activity was reviewed by CDC, deemed not research, and was conducted consistent with applicable federal law and CDC policy.^††^

## Results

Approximately 59.8 million bivalent COVID-19 doses were administered in the United States during September 1, 2022–September 30, 2023, including 40.5 million (67.7%) doses administered by FRPP partners. A total of 694 records were excluded from the FRPP database and 113 from the all-provider database because age data were invalid.

By age group, the highest percentage of COVID-19 bivalent doses administered by FRPP partners was to persons aged 18–49 years (70.6%), and the lowest was to children aged 6 months–4 years (5.9%) (Table 1). More than two thirds of males (66.9%) and females (68.6%) were vaccinated through FRPP partners. By racial and ethnic group, the highest proportions of bivalent COVID-19 doses administered by FRPP partners were among Asian (60.2%) and White persons (56.2%), and the lowest proportions were among AI/AN persons (21.9%) and persons of other races (22.3%). The percentage of doses administered to persons with unknown race and ethnicity was higher in the FRPP data (29.4%) than in the all-provider data (10.9%).

**TABLE 1:** Percentage of COVID-19 bivalent vaccinations administered by Federal Retail Pharmacy Program partners and recipient sex, age group, and race and ethnicity — United States, September 1, 2022–September 30, 2023

Characteristic	No. of bivalent vaccine doses administered by all providers	No. of bivalent vaccine doses administered by FRPP partners (%)
Total bivalent doses*	**59,776,140**	**40,458,857 (67.7)**
**Sex** ^†^		
Female	32,608,792	22,377,327 (68.6)
Male	27,010,446	18,071,154 (66.9)
**Age group**		
6 mos–4 yrs	600,238	35,114 (5.9)
5–11 yrs	1,752,601	588,776 (33.6)
12–17 yrs	2,141,050	1,158,364 (54.1)
18–49 yrs	15,791,006	11,144,137 (70.6)
50–64 yrs	13,765,721	9,709,461 (70.5)
≥65 yrs	25,725,524	17,823,005 (69.3)
**Race and ethnicity** ^§,¶^		
AI/AN	459,135	100,393 (21.9)
Asian	4,481,430	2,697,529 (60.2)
Black or African American	4,198,748	1,946,903 (46.4)
NH/OPI	134,374	74,571 (55.5)
White	35,294,990	19,826,869 (56.2)
Hispanic or Latino	5,874,775	3,267,637 (55.6)
Other race, NH	2,836,832	632,645 (22.3)

**Abbreviations:** AI/AN = American Indian or Alaska Native; FRPP = Federal Retail Pharmacy Program; NH = non-Hispanic; NH/OPI = Native Hawaiian or other Pacific Islander.

* A total of 694 records in the FRPP database and 113 in the all-provider database were excluded because of invalid age.

^†^ The sex of the recipient was unknown for 10,376 (0.03%) bivalent vaccine dose recipients reported in the FRPP data and 156,902 (0.26%) recipients reported in the all-provider data.

^§^ The race and ethnicity of the recipient was unknown for 11,912,310 (29.4%) FRPP dose recipients and 6,495,856 (10.9%) of all-provider dose recipients.

^¶^ Persons of Hispanic or Latino (Hispanic) origin might be of any race but are categorized as Hispanic; all racial groups are non-Hispanic.

Among the full analytic sample of 59 distinct jurisdictions, all but five (American Samoa, Northern Mariana Islands, Federated States of Micronesia, Marshall Islands, and Palau) had FRPP partners. Among the 54 jurisdictions with FRPP partners, at least one half of bivalent COVID-19 vaccine doses were administered by FRPP partners in 42 (77.8%) jurisdictions. Among the 52 jurisdictions with urban designated areas and the 48 jurisdictions with rural designated areas, the proportion of bivalent doses administered by FRPP partners was higher among urban areas (81.6%) compared with rural areas (60.0%) (Table 2). FRPP pharmacies administered ≥50% of bivalent doses in 45 of 52 (86.5%) jurisdictions’ urban designated counties and 33 of 48 (68.8%) jurisdictions’ rural designated counties.

**TABLE 2:** Percentage of COVID-19 bivalent vaccine doses administered by Federal Retail Pharmacy Program partners in urban and rural settings — United States, September 1, 2022–September 30, 2023

NCHS designation*	No. of bivalent vaccine doses administered by all providers	No. of bivalent vaccine doses administered by FRPP partners within geographic designations (%)
**Total bivalent doses administered** ^†^	**59,776,140**	**40,458,857 (67.7)**
Records excluded^§,¶^	8,943,617	64,371
Total analytic sample	50,832,523	40,394,486 (79.5)
**Administration setting**		
Urban	45,848,867	37,404,942 (81.6)
Rural	4,983,656	2,989,544 (60.0)

**Abbreviations:** FRPP = Federal Retail Pharmacy Program; NCHS = National Center for Health Statistics.

* The 2013 NCHS urban-rural classification scheme (https://www.cdc.gov/nchs/data_access/urban_rural.htm) was used to classify counties where bivalent doses were administered into urban and rural categories. Urban counties were defined by combining four of these six categories (large central metropolitan, large fringe metropolitan, medium metropolitan, and small metropolitan), and rural counties were defined based on two categories (micropolitan and noncore).

^†^ A total of 694 records in the FRPP data and 113 records reported in the all-provider data were excluded because of invalid reported age.

^§^ A total of 0.2% of records in FRPP data, and 15% of all-provider data were excluded because of missing county or no NCHS designation.

^¶^ Although the overall analytic sample for this analysis was 59 jurisdictions, seven U.S. territories and freely associated states (i.e., American Samoa, Northern Mariana Islands, Federated States of Micronesia, Guam, the Marshall Islands, Palau, and the U.S. Virgin Islands) lacked urban-rural county-level designations. Among the remaining jurisdictions, all 52 contained urban designated areas; however, four jurisdictions (Delaware, District of Columbia, New Jersey, and Rhode Island) had no rural designations, leaving only 48 jurisdictions represented in the final rural analyses.

## Discussion

During September 1, 2022–September 30, 2023, FRPP partners administered 40.5 million bivalent COVID-19 doses, representing more than two thirds (67.7%) of all bivalent COVID-19 doses administered across the United States and its territories and freely associated states. In comparison, previous data show that FRPP partners administered 45% of monovalent doses during February 11, 2021–January 31, 2022 (*2*). Factors that might have contributed to the higher proportion of bivalent compared with monovalent COVID-19 vaccines doses administered by FRPP partners include a higher level of awareness of COVID-19 vaccine availability at pharmacies, ease of accessibility (e.g., extended hours of operation, walk-in and scheduled appointments, and geographically convenient locations), and fewer COVID-19 mass vaccination clinics during this period compared with the time when the original monovalent vaccine first became available (*2*). Although FRPP partners were effective in making COVID-19 vaccines widely accessible, differences in use of FRPP partner vaccination services was observed across age groups, racial and ethnic groups, sex, and urbanicity.

Despite the availability of COVID-19 vaccines through FRPP partners and from other vaccine providers, overall U.S. bivalent vaccination coverage was substantially lower than that of completed monovalent primary COVID-19 vaccination series. Data from CDC’s COVID Data Tracker reveal that as of May 11, 2023 (the end date of the public health emergency), 17% of the U.S. population had received the bivalent vaccine,^§§^ compared with 69.5% who had completed a primary series. Among persons considering bivalent vaccination, commonly reported barriers to receipt of bivalent COVID-19 vaccine have included being too busy or forgetting to get vaccinated and having concerns related to side effects, whereas the main concerns reported by persons reporting no intent to receive a bivalent vaccine were more often related to trust, belief that vaccination was not necessary, and concerns about safety. However, the results of surveys conducted in March and April 2023 indicate that fewer than 5% of respondents reported access issues of time or costs as concerns, suggesting that access was not a substantial contributor to low vaccination rates (*4*).

FRPP vaccinations were reported for all evaluated demographic groups, including all age groups. However, FRPP partners administered the highest proportion of bivalent vaccine doses to adults, with similar percentages of doses administered to adults in all age groups (range = 69.3%–70.6%). FRPP partners administered a lower proportion of bivalent doses to children aged 5–11 years (33.6%) and 6 months–4 years (5.9%). This difference in percentages of doses administered to adult and pediatric recipients is not unexpected: historically, more adults than children have received annual influenza vaccination at pharmacies (*5*). Surveys conducted during September 2021 found that parents reported a higher level of trust when vaccinating their child at their regular clinic (63%), compared with vaccination at 1) a local pharmacy (34%), 2) a clinic different from their regular one (30%), 3) school with the parent present (25%), 4) temporary mass vaccination clinic (25%), and 5) school without the parent present (15%) (*6*). Pharmacy administration of COVID-19 vaccination to children was possible in part because of the Public Readiness and Preparedness Act, which lowered the age at which children could be vaccinated at pharmacies to 3 years in all states, making COVID-19 vaccination accessible for some age groups not typically vaccinated at pharmacies in many states (*7*). Although FRPP helped during this public health emergency, pediatricians, health departments and federally qualified health centers were needed to ensure that young children had adequate access to COVID-19 vaccines. 

FRPPs administered a large proportion of COVID-19 bivalent doses to most racial and ethnic groups. However, the proportion was lower for AI/AN persons, a group that might have relied more on Indian Health Service facilities or other vaccine providers.

The FRPPs’ contribution to COVID-19 bivalent doses was higher among urban than rural counties. Possible reasons for this difference are the potential higher accessibility of pharmacies in urban areas, as well as the fact that independent pharmacies in rural areas might have been less likely to partner with the FRPP. In addition, factors such as the availability of bivalent COVID-19 vaccines in primary care settings or other settings could have affected the proportion of COVID-19 bivalent doses administered by FRPP partners located in urban versus rural areas (*8*,*9*). Further evaluations are needed to understand the factors contributing to differences in pharmacy provider vaccination among urban and rural residents.

### Limitations

The findings in this report are subject to at least four limitations. First, COVID-19 vaccination coverage estimates were not possible using data from this analysis because unique persons vaccinated could not be identified. Second, the age groups used to describe COVID-19 vaccination among younger children include those aged 6 months–4 years. However, FRPP data only include vaccinated children aged ≥3 years, unlike the all-provider data, which included vaccinations administered to children and infants as young as age 6 months. Third, the higher number and percentage of records with race and ethnicity reported as unknown in the FRPP data compared with those in the all-provider data might have resulted in less accurate representation and potential underestimation of FRPP contributions for some racial and ethnic groups. Finally, the National Center for Health Statistics Urban-Rural Classification was developed in 2013, and the urban-rural designations used likely affected these analyses. Several counties classified as rural in 2013 might no longer be rural. In addition, a larger percentage of records were removed from the all-provider data (15.0%) than from the FRPP data (0.2%) because of the lack of matching urban–rural classification. Both factors might have skewed the overall pharmacy contribution, particularly in examining the percentage of urban and rural doses administered by FRPP partners.

### Implications for Public Health Practice

FRPP partners were critical in ensuring access to bivalent COVID-19 vaccination services throughout the United States. This partnership could serve as a model to address vaccination services needs for administration of routinely recommended vaccines and potential future responses to vaccine-preventable disease emergencies. Further strategies to support improvement in race and ethnicity data collection and reporting, particularly in pharmacy settings, are needed to help guide public health practices. Although the public health emergency has ended, the need to ensure that the U.S. population has equitable access to all recommended vaccines, including COVID-19 vaccines, remains. FRPP demonstrated that partnering with pharmacies, in addition to other vaccine providers, can help accelerate vaccine access provision across the United States and address other potential infectious diseases-related public health emergencies.

## Footnotes

* Providers were required by CDC to document COVID-19 vaccination in their medical records within 24 hours of administration and in their jurisdiction’s immunization information system (IIS) within 72 hours of administration during the public health emergency. IISs are confidential, computerized, population-based systems that collect and consolidate vaccination data from providers in 64 public health jurisdictions and can be used to track vaccines administered across multiple provider types and measure vaccination coverage. The 64 jurisdictions comprise the 50 U.S. States, eight U.S. territories and freely associated states (i.e., American Samoa, Northern Mariana Islands, Federated States of Micronesia, Guam, Marshall Islands, Palau, Puerto Rico, and the U.S. Virgin Islands), and six local jurisdictions (Chicago, Illinois; Houston, Texas; New York, New York; Philadelphia, Pennsylvania; San Antonio, Texas; and Washington, D.C.).

† Although there are a total of 64 IIS jurisdictions, the analytic sample for this report only includes 59 distinct jurisdictions because of local jurisdictional data (i.e., Chicago, Houston, New York City, Philadelphia, and San Antonio) being included within their respective state’s data.

§ Bivalent vaccine dose administration data from Idaho and Texas (from persons aged<18 years) were not reported to CDC through their IISs; instead, these jurisdictions submitted their data on bivalent vaccine doses administered using aggregate table shells aligned with the all-provider data for inclusion in the overall data.

¶ The 2013 National Center for Health Statistics (NCHS) urban-rural classification scheme (https://www.cdc.gov/nchs/data_access/urban_rural.htm) was used to classify counties where bivalent COVID-19 vaccine doses were administered in urban and rural categories. Urban counties were defined by combining four of these six categories (large central metropolitan, large fringe metropolitan, medium metropolitan, and small metropolitan), and rural counties were defined based on two categories (micropolitan and noncore). Records with missing administration counties and those lacking NCHS designations were excluded from the urban-rural analyses.

** The overall analytic sample for this analysis was 59 jurisdictions; however, seven U.S. territories and freely associated states (i.e., American Samoa, Northern Mariana Islands, Federated States of Micronesia, Guam, Marshall Islands, Palau, and the U.S. Virgin Islands) lacked urban-rural county level designations. Among the remaining jurisdictions, all 52 contained urban-designated areas; however, four jurisdictions (D.C., Delaware, New Jersey, and Rhode Island) had no rural designations, leaving only 48 jurisdictions represented in the final rural analyses.

†† 45 C.F.R. part 46.102(l)(2); 21 C.F.R. part 56; 42 U.S.C. Sect. 241(d); 5 U.S.C. Sect. 552a; 44 U.S.C. Sect. 3501 et seq.

§§
https://covid.cdc.gov/covid-data-tracker/#vaccinations_vacc-total-admin-rate-total
